# Quinic acid: a potential antibiofilm agent against clinical resistant *Pseudomonas aeruginosa*

**DOI:** 10.1186/s13020-021-00481-8

**Published:** 2021-08-06

**Authors:** Lan Lu, Yuting Zhao, Guojuan Yi, Mingxing Li, Li Liao, Chen Yang, Chihin Cho, Bin Zhang, Jie Zhu, Kun Zou, Qiang Cheng

**Affiliations:** 1 Key Laboratory of Medicinal and Edible Plants Resources Development of Sichuan Education Department, Sichuan Industrial Institute of Antibiotics, School of Pharmacy, Chengdu University, Chengdu, Sichuan People’s Republic of China; 2 Laboratory of Molecular Pharmacology, Department of Pharmacology, School of Pharmacy, Southwest Medical University, Luzhou, Sichuan People’s Republic of China

**Keywords:** Plant extracts, *Lonicerae Japonicae Flos*, Antibiofilm agents, Quorum sensing, Clinical resistant *Pseudomonas aeruginosa*

## Abstract

**Background:**

The biofilm state of pathogens facilitates antimicrobial resistance which makes difficult-to-treat infections. In this regard, it has been found that the compounds screened from plant extracts represent one category of the most promising antibiofilm agents. However, the antibiofilm activities and the active ingredients of plant extracts remain largely unexplored. In this background, the study is (1) to screen out the plant extracts with antibiofilm ability against *Pseudomonas aeruginosa*, and (2) to identify the active ingredients in the plant extracts and elucidate the underlying mechanism of the antibiofilm activities.

**Methods:**

Micro-broth dilution method, in vitro biofilm model, LC–MS/MS analysis and *P. aeruginosa*-mouse infection model were adopted to assess the antibiofilm activity. GC–MS analysis was performed to detect the active ingredients in plasma. RNA-Seq, GO analysis, KEGG analysis and RT-qPCR were adopted to elucidate the underlying mechanism of antibiofilm activities against *P. aeruginosa.*

**Results:**

*Lonicerae Japonicae Flos* (LJF) among 13 plants could exert significant inhibitory effects on bacterial biofilm formation, mobility and toxin release in vitro, and it could exert antibiofilm effect in vivo too. Moreover, quinic acid, as one metabolite of chlorogenic acid, was found as an active ingredient in LJF against the biofilm of *P. aeruginosa*. The active ingredient significantly inhibited EPS secretion in biofilm formation and maturity and could achieve synergistic antibiofilm effect with levofloxacin. It reduced the biofilm formation by regulating core targets in quorum sensing system. In GO process, it was found that the core targets were significantly enriched in multiple biological processes involving locomotion, chemotaxis and motility mediated by flagellum/cilium, which was related to KEGG pathways such as bacterial chemotaxis, oxidative phosphorylation, ribosome, biofilm formation, cyanoamino acid metabolism and quorum sensing. Finally, the binding of quinic acid with core targets rhlA, rhlR and rhlB were validated by molecular docking and RT-qPCR.

**Conclusions:**

In summary, the study verified the in vitro and in vivo antibiofilm effects of LJF against *P. aeruginosa* and elucidated the active ingredients in LJF and its conceivable pharmacological mechanism, indicating that quinic acid could have the potential of an antibiofilm agent against *P. aeruginosa* and related infections.

**Graphic abstract:**

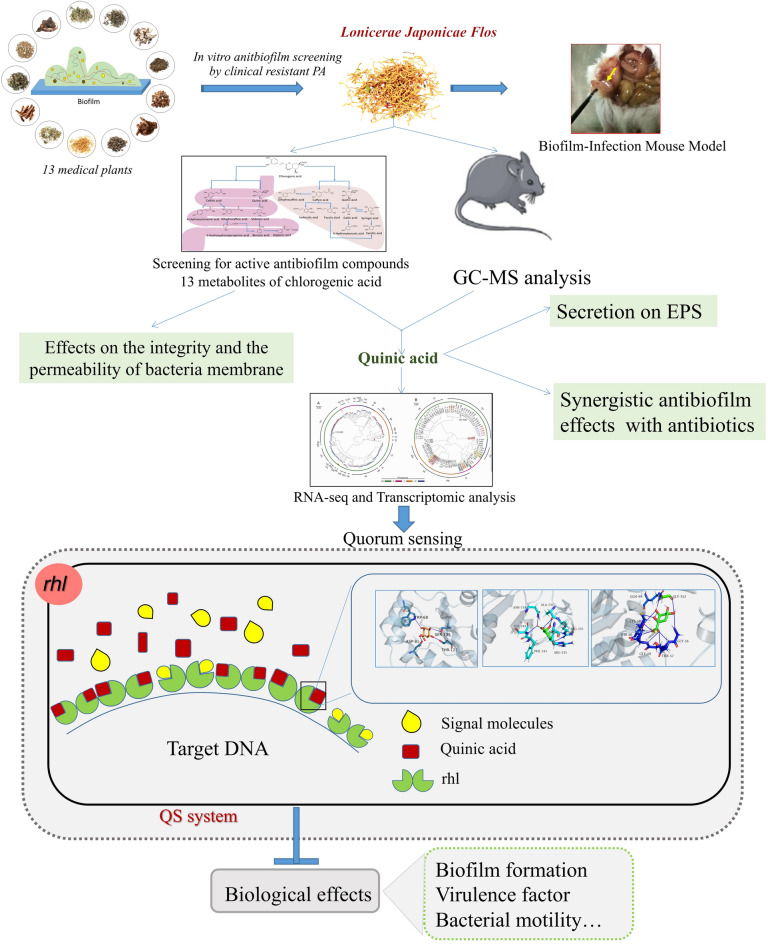

**Supplementary Information:**

The online version contains supplementary material available at 10.1186/s13020-021-00481-8.

## Introduction

Antimicrobial resistance (AMR) claims at least 6 million lives every year and poses a great threat to human health [1, 2]. It makes difficult-to-treat infections in which over 80% of microbial infections in the host are connected with pathogens existing in biofilm state that facilitates AMR, serious chronic infections, immune responses and external stress [3–5]. Among these pathogens, *Pseudomonas aeruginosa* (PA), a well-documented Gram-negative opportunistic pathogen which is notoriously inclined to form the surface-attached biofilm, has developed as one of main causative contributors of nosocomial infections based on its diverse genotypes and phenotypes. Therefore, novel anti-infective therapeutics and agents are urgently demanded to handle AMR and biofilm-related infections [6]. It is believed that biofilm formation as well as quorum sensing (QS) systems represent highly attractive targets for the development of such novel therapeutics and agents [7–9].

Currently, an explosive amount of studies are being conducted to discover antibiofilm agents and novel compounds against bacteria to develop drug resistance [10–12]. So far, numerous antibiofilm compounds have been discovered from the libraries of diverse plant extracts which represent the most promising candidates for screening of antibiofilm and anti-QS agents [13–15].

First of all, the study is to compare the ability of 13 plant extracts to modulate QS-dependent biofilm formation against clinical PA strains. Then, it will conduct a systematic investigation for the antibiofilm activities of plant extracts against PA, i.e., (1) testing their effects on motility, virulence factors and Acylated Homoserine Lactone (AHL) production and verifying the antibiofilm effects in a biofilm-infection mouse model; (2) identifying the active antibiofilm compounds/metabolites; (3) determining the potential pathways and targets of active antibiofilm compounds/metabolites by RNA-Seq and transcriptomic analysis as well as molecular docking so as to preliminarily understand the underlying mechanism of antibiofilm activities in the plant extracts.

## Materials and methods

### Strains and growth conditions

*Staphylococcus aureus* (ATCC 25923), *Escherichia coli* (ATCC 25922), PA *PAO1* (ATCC 27853), *Enterococcus faecium* ATCC 29212 and clinical isolates including PA (96 strains), Methicillin-sensitive *Staphylococcus aureus* (MSSA, 5 strains), Methicillin-resistant *Staphylococcus aureus* (MRSA, 5 strains), non extended-spectrum β-lactamase-producting *Escherichia coli* (*E. coli* E−, 5 strains), extended-spectrum β-lactamase *Escherichia coli* (*E. coli* E + , 5 strains), *Klebsiella pneumoniae* (KPN E- and KPN E+, 5 strains, respectively), *Acinetobacter baumannii* (ISAB, 5 strains), *Enterococcus faecalis* (EF, 5 strains), and *Enterococcus faecium* (AREF, 5 strains) were used in this study. All strains were kept in our laboratory and propagated in LB medium (1% tryptone, 0.5% yeast extract, 1.0% NaCl) at 37 °C unless otherwise specified.

### Plant extract preparation

Thirteen plants used in this study were bought from the store of Chinese medicine in Sichuan, China, including *Violae Herba, Smilacis Glabrae Rhizoma, Patriniae Herba, Prunellae Spica, Forsythiae Fructus, Scrophulariae Radix, Taraxaci Herba, Cremastrae Pseudobulbus, Paridis Rhizoma, Lysimachiae Herba, Lobeliae Chinensis Herba, Lonicerae Japonicae Flos* (LJF)*, Rabdosiae Rubescentis Herba*. Plant extracts were prepared according to the method described previously [16–18]. The plants were ground to powders using a mechanical grinder and then were extracted by maceration in water. In brief, 10 g of the powdered materials were mixed with 200 mL of water and boiled for 3 × 60 min. The solvent was filtered through Whatman filter paper No. 1 and concentrated on a rotary vacuum evaporator. The crude extract was reconstituted in water to make a stock solution of 1 g/mL. All the samples were stored at − 20 °C until use. Then, the plant extract with antibiofilm effect was qualitatively and quantitatively analyzed by ultra-high-performance liquid chromatography coupled with quadrupole time-of-fight mass spectrometry and high-performance liquid chromatography, respectively.

### Antibacterial activity and QS-controlled phenotypic assays

#### The susceptibility assay for stains (MIC values)

The minimum inhibitory concentration (MIC) using the broth micro-dilution method according to Clinical and Laboratory Standard (CLSI, 2015) were tested in three assays to test antibacterial activities including (1) levofloxacin against 96 clinically isolated strain of PA; (2) 13 plant extracts against PA1803 and ATCC 27853; (3) Chlorogenic acid (CA) and its metabolites including quinic acid, caffeic acid, p-coumaric acid, dihydrocaffeic acid, shikimic acid, benzoic acid, hippuric acid, isoferulic acid, ferulic acid, gallic acid, p-hydroxybenzoic acid, syringic acid, vanillic acid (Shanghai yuanye biotechnology Co. Ltd) against PA, MSSA, MRSA, *E. coli* E+, *E. coli* E−, KPN E−, KPN E+, ISAB, EF, and AREF (5 strains, respectively). Chemicals (analytical standard, purity > 99%, Sigma-Aldrich Trading Co. Ltd.) were dissolved in sterile water or DMSO. The same amount of DMSO served as the negative control. Briefly, two-fold dilutions of chemicals were prepared with LB medium in tubes and each tube was inoculated with 500 μL overnight bacteria culture, approximately 5 × 10^5^ CFU/mL. Then, the tubes were incubated at 37 °C for 24 h. The MIC was defined as the lowest concentration (mg/mL) of chemicals that completely inhibited visible growth of bacteria.

#### Biofilm inhibition assay

The biofilm inhibition assay was performed in 96-well flat-bottom polystyrene plates (Corning, USA) as described previously with some modifications [20]. Briefly, overnight cultures of PA1803 (OD_600 nm_ = 0.5) were diluted 1:100 into 200 μL of trypticase soytone broth and then incubated with plant extracts at 37 °C for 24 h without agitation. After cultivation, planktonic cells were removed with three washings, and then biofilms were stained with crystal violet (0.05%) for 20 min. Excess crystal violet was removed by distilled water and bound crystal violet was dissolved in 200 μL of 95% ethanol. Biofilms were quantified by reading the microplates at 570 nm.

#### Effect on virulence factors and motility in PA

The assays were performed as described previously [20]. For determining elastase activity, PA with different concentrations of LJF (1/4MIC, 1/8MIC, 1/16MIC) was grown at 37 °C for 18 h and then sterile supernatant (100 μL) was mixed with elastin Congo red (ECR, Sigma-Aldrich) buffer (900 μL, 1 mM CaCl_2_, 100 mM Tris, pH 7.5) containing 20 mg of ECR and incubated at 37 °C for 5 h. After incubation, the absorbance of the supernatant was determined at 495 nm. For pyocyanin determination, PA samples treated at previous concentrations (1/4MIC, 1/8MIC, 1/16MIC) of LJF were collected. The culture supernatant was extracted by chloroform and the chloroform layer was mixed with 2 M HCl. After 10 min centrifugation at 4 °C, the HCL layer was collected and measured at OD_520 nm_ to determine the production of pyocyanin. Rhamnolipids were determined according to the orcinol method. Briefly, 1 mL ether was added to 600 μL cell supernatant for rhamnolipid extraction. The ether layer was dried in the fume cupboard, and residuals were dissolved in 100 μL distilled water, 100 μL orcinol, and 700 μL H_2_SO_4_ (70%) were added to dissolve the rhamnoplipid. Absorbance at OD_420 nm_ was measured for quantification. For detection of alginate production, 70 μL of sterile supernatant at previous concentrations of LJF was mixed with 600 μL of boric acid/H_2_SO_4_ (4:1, v/v) for detecting alginate generation and vortexed. Then, 20 μL of 0.2% carbazole solution was added, and then incubated at 55 °C for 30 min. Absorbance at OD_530 nm_ was measured for alginate production. In motility inhibition assay, 5 μL of overnight PA cultures (OD_600nm_ = 0.5) were inoculated with different concentrations of LJF at the center of the swimming agar (1% tryptone, 0.5% NaCl, 0.3% agar, pH 7.2) and swarming agar medium (1% tryptone, 0.5% NaCl, 0.5% glucose, 0.3% agar, pH 7.2), respectively. Plates were cultivated at 37 °C overnight. The swarming motility was determined by the diameter of turbid zone.

#### Growth curve analysis

Effects of LJF and CA on the growth of PA were determined by growth curve analysis. Briefly, 100-fold diluted overnight bacterial cultures were added to the sterile tubes and mixed with different concentrations of LJF or CA (MIC, 1/2MIC, 1/4MIC, 1/8MIC, 1/16MIC, 1/128MIC). LB medium and LB with 5% DMSO (the same DMSO concentration as the MIC sample) were used as negative control. The tubes were incubated at 37 °C. The absorbance at OD_600 nm_ was measured at time points.

#### Biofilm-infection mouse model

The model was performed as described previously with some modifications [21, 22]. The biofilm of PA was grown on catheters (length = 0.5 cm, *r* = 0.4 cm). Each catheter was placed into a 5 mL tube containing 3 mL LB for culture. 100 µL PA1803 suspension at 0.5 McFarland which includes 1.5 × 10^7^ bacteria cells was inoculated into each tube (1.5 × 10^6^ cfu/mL) and cultured for 3 days at 37 °C. Thirty male mice, weighing 18–22 g, were anesthetized with intraperitoneal injection of 4% chloral hydrate at 0.01 mL/g. This Animal experiment in this project had been approved by the Laboratory Animals Ethics Committee of Chengdu University. Then the catheters with biofilms were rinsed with sterile physiological saline solution and implanted at abdominal wall. Mice were randomly divided into three groups with 10 mice in each group. After implantation, LJF (1/4MIC and MIC) were immediately injected intragastrically every day. At the end of antibiofilm therapy (7 days after), the implanted catheters and peripheral tissue in all four groups were taken out to examine the bacterial counts in biofilms. Hematoxylin–eosin (HE) staining was used to evaluate the degree of tissue inflammation.

#### 260 nm release assay

The assay were conducted as previously reported [23]. The bacterial suspension was separated into several flasks. A certain amount of the gallic acid or dihydrocaffeic acid was added to each flask except the control. Samples of 1.5 mL were removed from the flasks every 20 min. The samples were then immediately filtered with 0.2 mm syringe filters to remove the bacteria. The supernatant was then diluted appropriately and optical density at 260 nm was recorded.

#### Determination of bacteria liquid conductivity

The method was performed as previously reported [24]. PA1803 grown to logarithmic phase were washed three times with 0.2 mol/L phosphate buffer (pH = 7.4). Bacterium liquid concentration was adjusted to 1 × 10^6^ CFU/mL, and 5 mL of sample was taken to mix with MIC of dihydrocaffeic acid/gallic acid. Conductivity was measured every 10 min. Sterile water was then used to replace dihydrocaffeic acid/gallic acid for experiments as blank control.

#### Effect on EPS secretion

EPS was extracted as previously reported [25–27]. Briefly, PA1803 suspensions (1%, v/v) were transferred to the fresh culture medium, and treated with quinic acid (1/16MIC, 1/8MIC, 1/4MIC and 1/2MIC). The suspensions were centrifuged to collect the supernatant. The EPS in the supernatant was then precipitated in 80% (v/v) cold ethanol. Then the supernatant was removed after centrifugation at 4 °C. Finally, 1 mL of 0.5% phenol and 5 mL of sulfuric acid were added to each group at the same rate for polysaccharide precipitation. Changes in EPS could be quantified by UV spectrophotometry through 490 nm wavelength.

#### Synergistic antibiofilm effects

The synergistic antibiofilm effects of quinic acid with two macrolides (clarithromycin and azithromycin) and levofloxacin at sub-MICs (1/64MIC, 1/32MIC, 1/16MIC, 1/8MIC, 1/4MIC 1/2MIC and MIC) were tested using the biofilm inhibition assay.

### Analytical methods

#### LC–MS/MS analysis

To assess the influence of LJF on the levels of C_4_-HSL and 3-oxo-C_12_-HSL (Sigma-Aldrich) in PA1803, the overnight cultures of PA1803 were diluted 1:1000 into 50 mL of LB with LJF (1/16MIC, 1/4MIC and MIC) and incubated at 37 °C at 200 rpm for 72 h. The supernatant was extracted three times using acidified ethyl acetate (1:1, v/v). The solvent was evaporated under reduced pressure and residues were dissolved in methanol. The levels of AHLs were determined using LC–MS/MS as described previously [19]. AHLs were analyze using Agilent 64d10B system (Agilent 1200 high performance liquid chromatograph equipped with Agilent Poroshell 120 SB-C18 column (4.6 mm × 100 mm, 2.7 mm). Agilent g1367c autosampler, Agilent g6410b triple Quad LC–MS/MS mass spectrometer and Agilent mashhunter workstation software version b.03.01 mass spectrometry data processing software, Agilent company of the United States). Briefly, peaks corresponding to C_4_-HSL and 3-oxo-C_12_-HSL were detected based on their MS/MS fragment ions and the retention time of AHLs standards. The area of the ion *m/z* 102 was used to quantify each AHL due to its specificity and better signal-to-noise ratio. Peak area calculation was performed by the extracted ion chromatograms.

#### GC–MS analysis

The quinic acid was accurately weighed and dissolved in acetonitrile to obtain the internal standard stock solution (4 μg/mL), which was stored at 4 °C. Blood was taken from the tail vein of rats at 0.5, 1, 2, 4, 6 and 24 h after oral administration of 6 g/kg LJF, and plasma was prepared by centrifugation for 20 min at 4 °C and 1000 g. 200 μL plasma was precipitated with 100 μL acetonitrile and centrifuged for 5 min and 6000*g*. The supernatant was dried with nitrogen. The solid is dissolved in 100 μL acetonitrile and 100 µL BSTFA + TMCA (99:1). The reaction was induced at 60 °C for 1 h. After reaction, the sample was centrifuged at 12,000*g* for 5 min. Then, supernatant was transferred into the vial for GC–MS analysis. The injection volume was 1 μL. GC was conducted with the Clarus 680 system (PerkinElmer, USA), consisting of an auto-sampler. Samples were separated by Elite-5 MS column (30 m × 0.25 mm, 0.25 μm) (PerkinElmer, USA), and were detected using the Clarus SQ 8 MS (PerkinElmer, USA) with electron impact ion (EI) source. The fragmentation patterns of mass spectra were compared with those stored in the spectrometer database using the National Institute of Standards and Technology Mass Spectral database (NIST17). The carrier gas was high purity helium (purity ≥ 99.999 × 10–2 (v/v)). The instrument was set to an initial temperature of 50 °C, and maintained at this temperature for 3 min. At the end of this period, the oven temperature was raised upto 180 °C, at the rate of an increase of 8 °C/min, then raised up to 280 °C at the rate of an increase of 10 °C/min maintained for 5 min. Injection port temperature was ensured as 250 °C and Helium flow rate as 1 ml/min. Samples were analyzed in a positive ionization pattern and under total ion monitoring (TIC) mode with the following optimized MS conditions: inlet line temperature, 200 °C; source temperature, 220 °C; multiplier, 1600 V. Under these conditions, the retention time of CA and quinic acid was 22.034 min and 32.799 min, respectively.

#### RNA-seq and transcriptomic analysis

Overnight PA culture was diluted 1:100 to achieve 5 × 10^8^ bacteria cells/mL and 100 mL of diluted culture was incubated to OD_600 nm_ = 0.4–0.6. The cells were then challenged with 30 mg/mL LJF for 24 h and collected for total RNA isolation using TRIzol reagent (Invitrogen, USA). Library construction and Illumina NextSeq 500 platform-based RNA-Seq were performed by Novogene Co., Ltd. (Beijing, China). Sequencing libraries were generated using NEBNext® UltraTM RNA Library Prep Kit for Illumina® (NEB, USA) following manufacturer’s recommendations and index codes were added to attribute sequences to each sample. Reads were mapped to the PA PAO1 genome (GenBank accession number: AE004091) using Bowtie 2 (version 2.2.4). Subsequently, Rockhopper software was used to calculate the values of differentially expressed genes (DEGs) using RPKM (reads per kilobase per million reads). Genes with an adjusted P-value < 0.05 found by DESeq were assigned as differentially expressed. Genes were annotated by searching the Kyoto Encyclopedia of Genes and Genomes (KEGG) and the Gene Ontology (GO) databases.

#### Quantitative real-time PCR

Quantitative real-time polymerase chain reaction (qRT-PCR) was performed to detect levels of QS-related genes. PA was grown in LB medium supplemented with or without LJF (1/4 MIC) at 37 °C at 200 rpm for 24 h. Samples were lysed in Trizol reagent. Total RNAs were isolated and 2 mg RNA was reversely transcribed using RevertAid Reverse Transcriptase (Thermo Scientific). For detection of gene expression, 2 SYBR Green reagent, 0.5 mL cDNA together with primers at the concentration of 0.25 mM were added to a 384-well plate for qPCR analysis. Quantitative PCR was performed on the ViiATM7 real-time PCR system (Applied Biosystems) with rpoD used as an internal control. Gene expression was analyzed by the comparative CT method and expressed as compared to rpoD.

**Table1:** Core targets of antibiofilm activity of quinic acid against PA

Uniprot ID	Gene	Protein	Hydrogen bonds	Affinity score
P54292	rhlR	Regulatory protein RhlR	5 hydrogen bonds (Typ 68, Asp 81, Thr 121 and Ser 135)	− 6.4
Q51559	rhlA	3-(3-hydroxydecanoyloxy)decanoate synthase	7 hydrogen bonds (Asn 219, Ala 231, Arg 232 and Ser 244)	− 5.7
Q9HXE5	rhlB	ATP-dependent RNA helicase RhlB	10 hydrogen bonds Gly 56, Thr 57, Gly 58, Lys 59, Thr 60, Gln 99 and Gly 352)	− 5.6

#### Molecular docking

The sequence of rhlR, rhlA and rhlB was obtained from UniProt database and SWISS-MODEL database was used to construct the 3D structures of rhlR, rhlA and rhlB. The structure of quinic acid in sdf format was downloaded from PubChem [28] (https://pubchem.ncbi.nlm.nih.gov/). Docking of quinic acid with rhlR, rhlA and rhlB structure was performed using AutoDock vina [29].

### Statistical analysis

The results are expressed as mean ± SEM. One-way analysis of variance (ANOVA) was performed for comparing differences between groups followed by the Tukey's test. P value below 0.05 was considered statistically significant.

## Results

### In vitro screening of 13 plant extracts with antibiofilm activities against clinical resistant isolates of PA

**Fig. 1:**
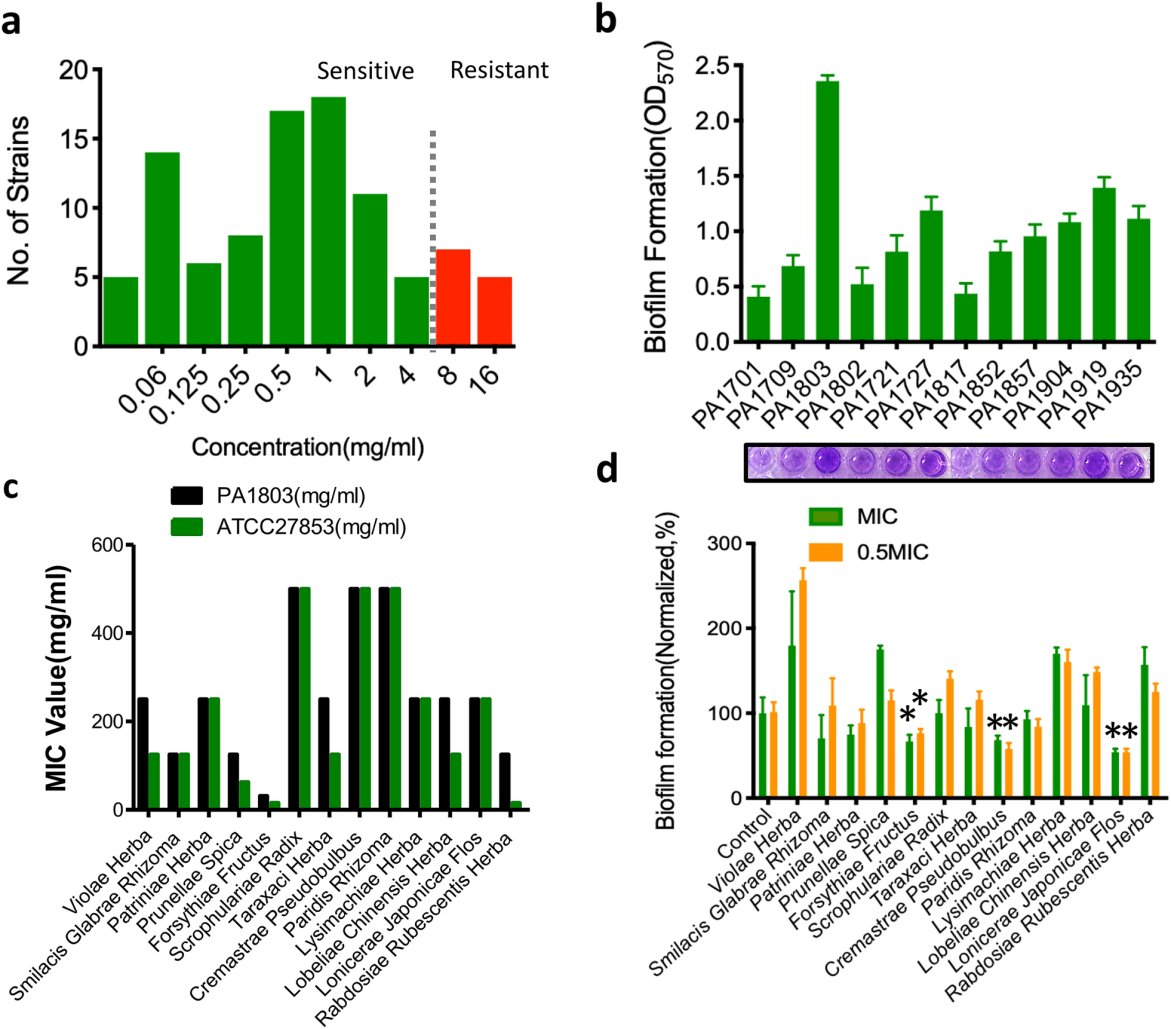
In vitro screening of 13 plant extracts with antibiofilm activities against clinical resistant isolates of PA. **a** The distribution of MIC values against levofloxacin of 96 clinical isolates of PA. **b** The level of biofilm formation in 12 resistant strains of PA. **c** The MIC values of 13 plant extracts against PA1803 and ATCC 27853. **d** The antibiofilm activities of 13 plant extracts against PA1803 at MIC and 1/2MIC. *P < 0.05, compared with corresponding control

The screening for clinically resistant isolates of PA and the testing of the levels of biofilm formation were first conducted in the study. See Additional file 1: Table S1 for the susceptibility (MIC values) of planktonic PA. Among the 96 clinical isolates, 84 strains were antibiotically susceptible while 12 strains with MIC values ≥ 8 μg/ml were antibiotically resistant (Fig. 1a). The level of biofilm formation in 12 resistant strains was tested by the in vitro biofilm model (crystal violet staining). One isolate numbered PA1803 with the highest level in biofilm formation (Fig. 1b) was selected to establish the biofilm model for in vitro screening of antibiofilm activity of 13 plant extracts.

First, the MIC values of 13 plant extracts against PA1803 were tested and compared with those of ATCC 27853. The results showed that most of the MIC values of 13 plant extracts against PA1803 were higher than or equal to those against ATCC 27853 (Fig. 1c). Then, the effect on biofilm formation of PA1803 was tested at MIC and 1/2MIC to briefly assess antibiofilm profiles of 13 plant extracts. It was clear that the plant extracts including LJF, *cremastrae pseudobulbus* and *forsythiae fructus* had significantly decreased the level of biofilm formation (Fig. 1d).

### The effects of LJF on biofilm formation, motility, virulence factors and AHL production of PA1803

**Fig. 2:**
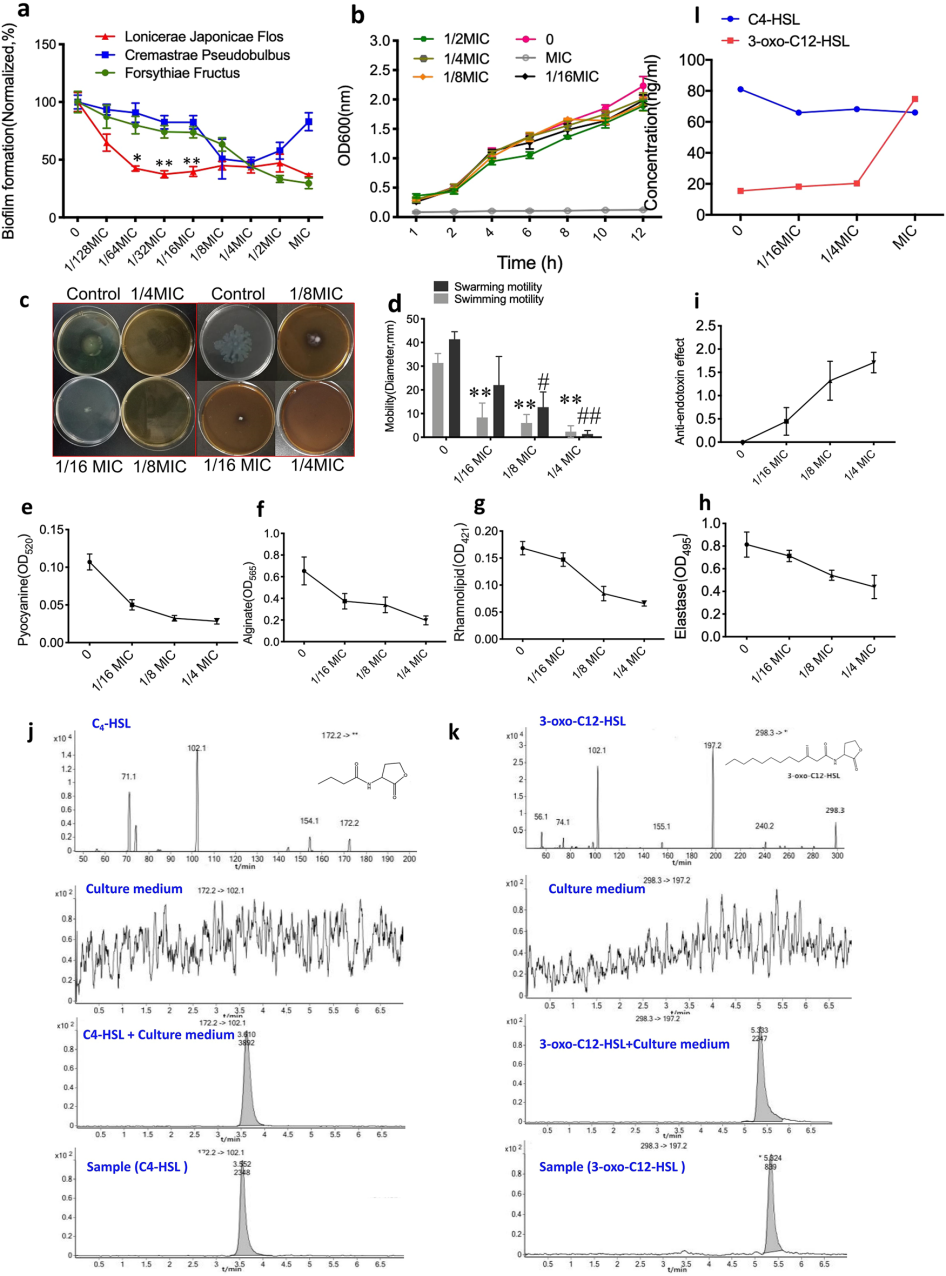
The effects of LJF on biofilm formation, motility, virulence factors and AHL production. **a** The dose–response curves of antibiofilm effects of LJF, *Cremastrae Pseudobulbus* and *Forsythiae Fructus*. **b** Growth curves of bacterial growth at different sub-MICs of LJF. **c**, **d** Swimming and swarming motility related to biofilm adhesion. **e**–**h** The virulence factors related to pathogenicity (pyocyanin, alginate, elastase and rhamnolipid). **i** Anti-endotoxin effects. **j**, **k** Product ion mass spectrometry of C_4_-HSL and 3-oxo-C_12_-HSL(1st line), culture medium (2nd line), culture medium + C_4_-HSL/3-oxo-C_12_-HSL (3rd line) and culture supernatants of PA treated with LJF (1/16MIC, 1/4MIC and MIC) for 72 h by LC–MS/MS analysis. l. Statistics analysis of C_4_-HSL and 3-oxo-C_12_-HSL level. *P < 0.05, **P < 0.01, ^#^P < 0.05, ^##^P < 0.01, compared with corresponding control

A series of sub-minimum inhibitory concentrations (sub-MICs) of the plant extracts including LJF, *cremastrae pseudobulbus* and *forsythiae fructus*, ranging from 1/128MIC to MIC, were selected to test the antibiofilm effect. The dose–response curves indicated that, at lower sub-MICs (1/64MIC to 1/16 MIC), LJF could exert the most potent antibiofilm action among the 3 plant extracts (*P < 0.05, **P < 0.01, Fig. 2a) and did not affect bacterial growth (Fig. 2b). The effects of LJF on the motility related to biofilm adhesion (Fig. 2c, d), the virulence factors related to pathogenicity (pyocyanin, alginate, elastase and rhamnolipid) as well as anti-endotoxin action were further investigated (Fig. 2e–i). The results showed that LJF had obvious inhibitory effects on motility and virulence factors. Two AHLs in QS system of PA1803 were also tested to evaluate the effect of LJF on them. LC–MS/MS analysis confirmed that two major AHLs, C_4_-HSL and 3-oxo-C_12_-HSL, were detectable in culture supernatants (Fig. 2j, k). Exposure to different concentrations of LJF (1/16MIC, 1/4MIC and MIC) for 72 h caused a slight decrease in both peak and area of C_4_-HSL but not in those of 3-oxo-C_12_-HSL (Fig. 2l). The findings revealed that LJF could exert significant inhibitory effects on bacterial biofilm formation, mobility and toxin release regardless of the AHLs of PA1803.

### Antibiofilm effect of LJF in the biofilm-infection mouse model

**Fig. 3:**
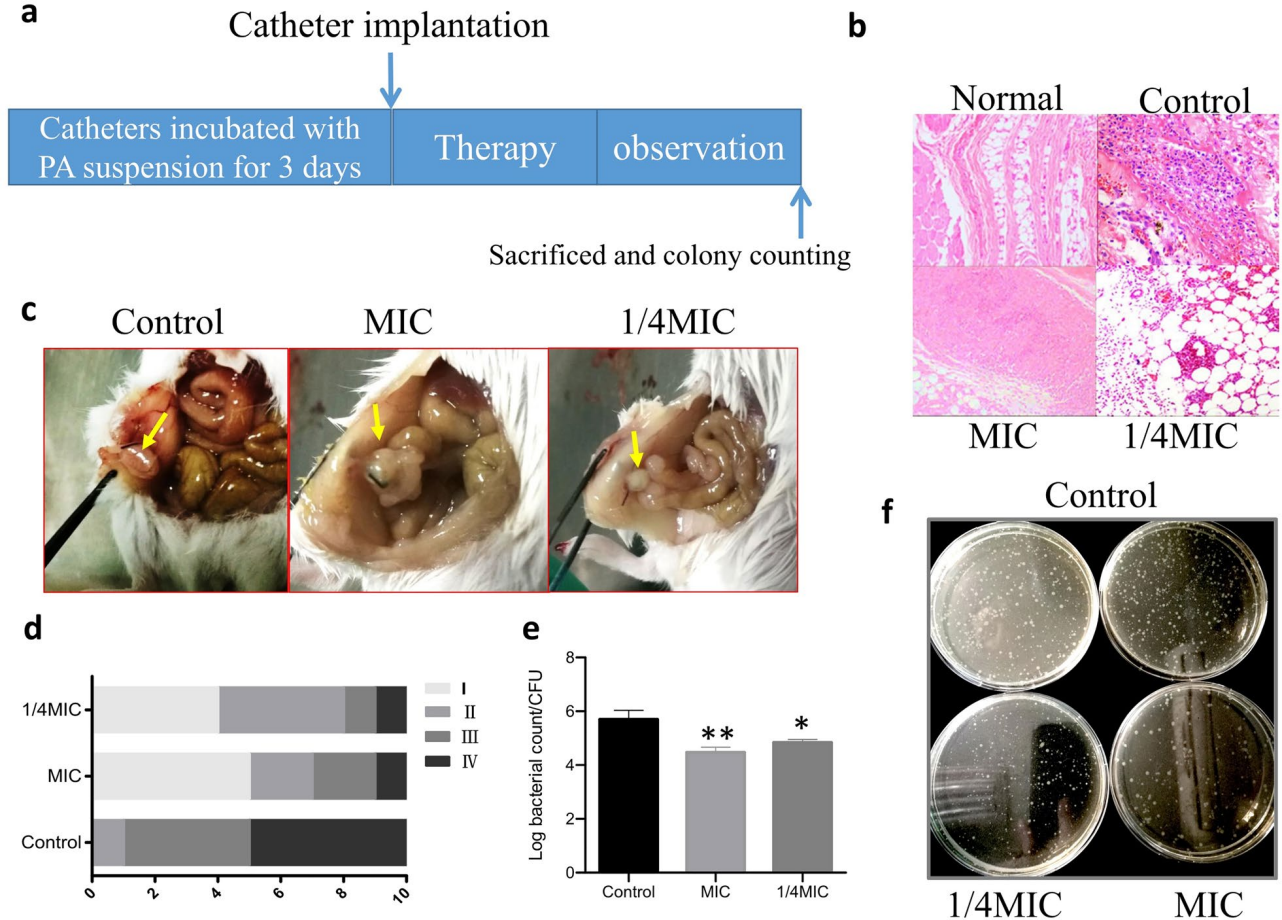
LJF exerting its antibiofilm effect in the biofilm-infection mouse model. **a** Schematic representation of treatment regimen in the biofilm-infection mouse model. **b** Peripheral tissue of implanted catheters by HE staining. **c** The representative pictures of pathological changes. **d** The pathological changes of three groups were compared. The incidence of pathological changes and the severity of inflammation were assessed by four grades: Grade I: normal; grade II: hyperemia and swelling; grade III: hyperemia, swelling and adhesion; grade IV: abscess, bleeding and adhesion. **e**, **f** The statistics analysis and the representative picture of colony counting in each group. *P < 0.05, **P < 0.01, compared with corresponding control

After the biofilm-infection mouse model was established (Fig. 3a), the mice were divided into three groups: the control group, the group treated with 1/4MIC of LJF and the group treated with MIC of LJF. Compared with the control group, the colony counting decreased in the groups treated with MIC and 1/4 MIC of LJF (Fig. 3e, f). It was also found that the incidence of pathological changes such as abscess, bleeding and inflammation in the groups treated with MIC and 1/4 MIC LJF decreased (Fig. 5c, d). Immunohistochemical results showed that the level of inflammatory cell infiltration of the two groups was relatively lower than that of the control group (Fig. 3b). It was concluded that the treatment with 1/4 MIC of LJF could exert an antibiofilm effect in vivo with lower colony counting and less pathological changes.

### Screening active ingredients from metabolites of CA in LJF

**Fig. 4:**
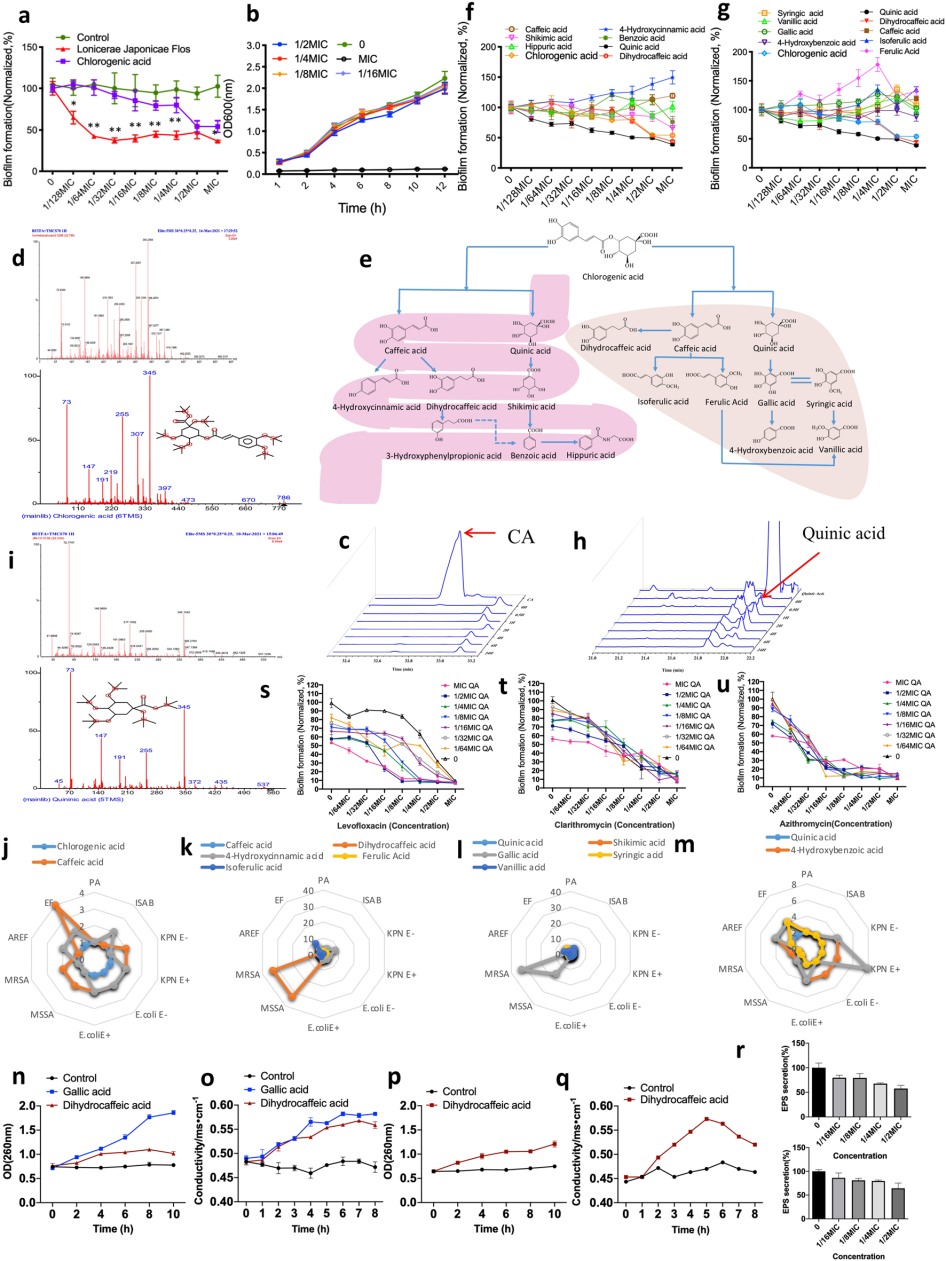
Active antibiofilm compounds from metabolites of CA in LJF. **a** Antibiofilm activity of LJF and CA. **b** Growth curves of bacterial growth at different sub-MICs of CA. **c** GC–MS analysis of CA at 0.5, 1, 2, 4, 6, 24 h. The red arrow indicates the location of the peak of CA. **d** Measured ion spectrum (upper) and matched ion spectrum in NIST17 database (lower) of CA. **e** Metabolic pathways of CA in intestine (left) and liver (right). **f** In vitro antibiofilm effects of CA and its intestinal metabolites. **g** In vitro antibiofilm effects of CA and its liver metabolites. **h** GC–MS analysis of quinic acid at 0.5, 1, 2, 4, 6, 24 h. The red arrow indicates the location of the peak of quinic acid. **i** Measured ion spectrum (upper) and matched ion spectrum in NIST17 database (lower) of quinic acid. **j** The antibacterial activity ratio of caffeic acid and quinic acid to CA. **k** The antibacterial activity ratio of caffeic acid and its main metabolites to CA. **l**, **m** The antibacterial activity ratio of quinic acid and its major metabolites to CA. **n**–**o** Effects of dihydrocaffeic acid and gallic acid on the integrity (n) and the permeability (o) of bacteria membrane against MRSA. **p**-**q** Effects of dihydrocaffeic acid on the integrity (**p**) and the permeability (**q**) of bacteria membrane against MSSA. **r** Effects of quinic acid on EPS secretion in biofilm formation (upper) and in mature biofilm (lower). **s**–**u** The synergistic antibiofilm effects of sub-MIC quinic acid with levofloxacin (**s**), clarithromycin (**t**) and azithromycin (**v**). QA: quinic acid. *P < 0.05, **P < 0.01, compared with corresponding control

Based on the results that LJF can exert antibiofilm activity in vitro and in vivo, the study attempted to elucidate the active antibiofilm ingredient in LJF. CA has been extensively reported as the main compound that possesses in vitro antibacterial and antibiofilm activities in various plants including LJF. Thus, the antibiofilm action of CA against PA1803 was first assessed compared with that of LJF (Fig. 4a). Interestingly, although CA was reported as the main antibacterial component in LJF, it showed only moderate antibiofilm action which was much lower than LJF at sub-MICs (*P < 0.05, **P < 0.01, Fig. 4a), without affecting bacterial growth (Fig. 4b). Therefore, it was speculated that some more effective compounds, other than CA, existed in LJF with significant antibiofilm effect.

Many literatures suggest that CA is rapidly metabolized into active ingredients in vivo, and the bioavailability of CA depends largely on its metabolites. To evaluate the antibiofilm activity of CA in vivo, the concentration of CA was first checked. GC–MS data showed that CA could not be found in plasma at all the time points after oral administration (Fig. 4c, d), which complied with the previous findings that CA was rapidly metabolized into active ingredients after entering the body. Thus, the metabolites of CA in intestinal (Fig. 4e, left) and liver (Fig. 4e, right) metabolic pathway were summarized to investigate their antibiofilm effects. Obviously, the metabolites of CA such as quinic acid and dihydrocaffeic acid could eliminate the biofilm produced by PA1803 at sub-MICs ranging from 1/64MIC-1/2MIC. In particular, at 1/128MIC-1/2MIC, quinic acid could exhibit more potent inhibitory effect on biofilm formation than CA *per se* (Fig. 4f, g). Thus, such CA metabolites as quinic acid and dihydrocaffeic acid, other than CA *per se*, could act as the active ingredients with antibiofilm effect in LJF at sub-MICs. Under the GC–MS conditions, quinic acid was detectable in plasma. It began to increase in plasma at 0.5 h and reached the highest concentration at 2 h after LJF administration (Fig. 4h, i).

It is reported that CA has antibacterial effect against both Gram-positive and Gram-negative bacteria [30–33]. In consideration of the antibiofilm activity of CA enhanced by its metabolites, further investigation was conducted for the antibacterial spectrum so as to compare the antibacterial effect of CA with its metabolites. Based on MIC values, the comparative study on the antimicrobial activities of CA and its 13 metabolites in vitro was performed against clinical strains including PA, MSSA, MRSA, *E. coli* E+, *E. coli* E−, KPN E-, KPN E+, ISAB, EF, and AREF (Fig. 4j–m). It should be noted that the molecular weight of CA was twice that of caffeic acid and quinic acid respectively, thus the antibacterial activity ratio of MIC_50 CA_/MIC_50 metabolite_ equal to 2 (with the same molar concentration) indicated the equivalent antibacterial effect. The antibacterial activity ratio of MIC_50 CA_/MIC_50 metabolite_ equal to or larger than 4 times indicated enhanced antibacterial effect. For most tested strains, the antibacterial activities of some main metabolites were enhanced, ranging from 4 to 33 times higher than CA *per se*. Benzoic acid, p-hydroxybenzoic acid, p-coumaric acid, dihydrocaffeic acid and gallic acid showed stronger antimicrobial activities than the other metabolites. Notably, the antibacterial action of dihydrocaffeic acid and gallic acid against MSSA and MRSA was increased 33 times respectively (Fig. 4j–m). Interestingly, the antibacterial activity against PA of all the metabolites of CA was not statistically different from that of CA *per se*, revealing that the metabolites of CA did not affect the bacterial growth of PA.

In summary, for most bacteria especially MSSA, MRSA, KPN E + and EF, the metabolites of CA such as dihydrocaffeic acid and gallic acid can broaden the antibacterial spectrum and enhance the antibacterial activity of CA in vivo. As for PA, the metabolites (eg. quinic acid) of CA mainly enhance the antibiofilm action other than the antibacterial action, which demonstrates better antibiofilm effect in vivo.

Thus, new experimental evidence was provided to investigate the anti-bacterial/antibiofilm effect of the metabolites of CA. Figure 4n–q showed the change in OD_260 nm_ values and conductivity of PA1803 after being treated by dihydrocaffeic acid/gallic acid at different times. However, compared with dihydrocaffeic acid, the difference of OD_260 nm_ between gallic acid and the control group was much larger, further indicating that gallic acid has stronger effect on the integrity of bacteria membrane. In the determination of permeability assayed by bacteria liquid conductivity, the conductivity of PA1803 treated by dihydrocaffeic acid/gallic acid showed no difference from that in the control group when the interaction time to PA1803 was less than 1 h. With an increase in interaction time, dihydrocaffeic acid and gallic acid gradually increased the conductivity of bacterial liquids, reflecting an increase in the membrane permeability. Figure 4r showed that quinic acid at some sub-MICs significantly inhibited EPS secretion in biofilm formation and mature biofilm of PA1803.

Obviously, the effects of dihydrocaffeic acid and gallic acid on the integrity and permeability of bacteria membrane were related to their antibacterial activity against MSSA and MRSA. Quinic acid exerted its antibiofilm action by interfering with EPS secretion.

Furthermore, the synergistic antibiofilm effects of quinic acid with two macrolides (clarithromycin and azithromycin) and levofloxacin at sub-MICs (1/64MIC, 1/32MIC, 1/16MIC, 1/8MIC, 1/4MIC 1/2MIC and MIC) were tested. Compare with the MIC levofloxacin, a series of novel combination of administration including 1/2MIC levofloxacin + 1/2 quinic acid, 1/2MIC levofloxacin + 1/4 quinic acid, 1/2MIC levofloxacin + 1/8 quinic acid, 1/4MIC levofloxacin + 1/2 quinic acid, 1/4MIC levofloxacin + 1/4 quinic acid, 1MIC levofloxacin + 1/8 quinic acid and 1/8MIC levofloxacin + 1/2 quinic acid showed no significant difference. The combination of levofloxacin and quinic acid at some lower sub-MICs such as 1/4MIC levofloxacin + 1/4 quinic acid and 1/4MIC levofloxacin + 1/8 quinic acid exerted similar antibiofilm effect as MIC levofloxacin did, indicating that quinic acid and levofloxacin at sub-MICs had synergistic antibiofilm effect. For two macrolides, clarithromycin or azithromycin *per se *at some sub-MICs showed significant antibiofilm effect. However, the combined administration of the macrolides and quinic acid showed limited synergistic antibiofilm effect.

### The mechanism of antibiofilm activity of quinic acid

**Fig. 5:**
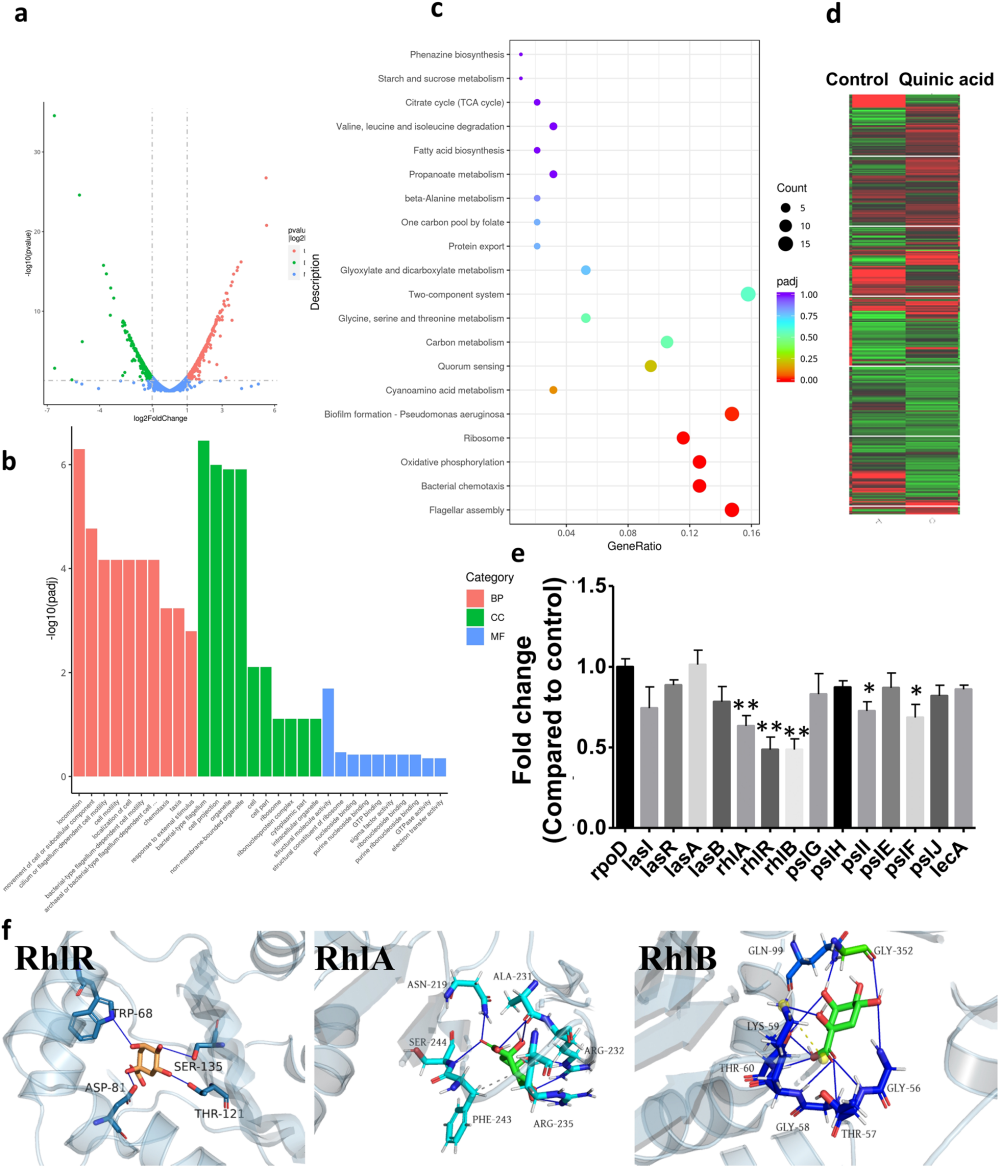
Characterizing the mechanism of antibiofilm activity of quinic acid. **a** Volcano plot displaying DEGs between the control group and the group treated with LJF at 1/4 MIC. **b** GO analyses for DEGs. BP Biological process, CC Cellular components, MF Molecular function. **c** KEGG pathway enrichment analysis of DEGs. **d** Hierarchical clustering of DEGs. **e** qRT-PCR validation for detecting the level of selected DEGs. **f** Computational docking of quinic acid binding to RhlR, RhlA and RhlB in PA*.* Blue line indicates hydrogen bonding. *P < 0.05, **P < 0.01, compared with corresponding control

To investigate the potential mode of antibiofilm action of quinic acid, further study was carried out to assess the effects of quinic acid on the level of QS-related genes and proteins. Changes in the level of QS-related genes in PA1803 treated with 1/4MIC of quinic acid were first assessed by RNA-Seq and transcriptomic analysis and validated by qRT-PCR. A total of 590 DEGs were assessed by RNA-Seq, including 255 down-regulated genes and 335 up-regulated genes, in PA1803 treated with quinic acid (Fig. 5a, d). The pathway enrichment analysis was executed to illuminate the underlying mechanism of antibiofilm activity of quinic acid against PA. The GO analysis demonstrated that the potential targets were mainly related to locomotion, movement of cell or sub-cellular component, cilium or flagellum-dependent cell motility, cell motility, localization of cell, bacterial-type flagellum-dependent cell motility (BP), bacterial-type flagellum, cell projection, organelle, non-membrane-bounded organelle (CC), structural molecule activity (MF), etc. The KEGG pathway enrichment analysis displayed that the potential targets were significantly enriched in flagellar assembly (pae02040), bacrerial chemotaxis (pae02030), oxidative phosphorylation (pae00190), ribosome (pae03010), biofilm formation-*Pseudomonas aeruginosa* (pae02025),cyanoamino acid metabolism (pae00460) and quorum sensing (02024), etc. (Fig. 5b, c). Subsequently, the level of some important QS-related DEGs, namely lasI, lasR, lasA, lasB, rhlA, rhlR, rhlB, pslG, pslH, pslI, pslE, pslF, pslJ and lecA, were validated by qRT-PCR (Fig. 5e). The most obvious downregulation was found in the level of rhlA, rhlR and rhlB related to biofilm formation and motility by approximately 50%-60% in PA1803 treated with quinic acid at 1/4MIC. Similarly, exposure to 1/4MIC quinic acid also caused a significant decrease in the level of pslI and pslF related to psl synthesis and transport in EPS secretion in PA1803 (Fig. 5e). The results in qRT-PCR were generally in accordance with those analyzed by RNA-Seq and transcriptomic analysis.

To investigate the interaction between quinic acid and the target proteins including RhlR, RhlA and RhlB in rhl system, 3D structures of these proteins were constructed. Figure 5f showed the 3D structures of RhlR, RhlA and RhlB binding with quinic acid predicted by AutoDock vina. The most favorable model had an affinity score of − 6.4, − 5.7, − 5.6 kcal/mol of the three target proteins (Table1). Previous studies had proved that a binding energy ≤  − 5.0 kcal/mol could imply a feasible binding capacity between the ligand and the receptor. The model showed that quinic acid fitted inside a pocket on the receptor structure and formed many hydrogen bonds with aromatic amino acid residues in the structures of RhlR, RhlA and RhlB, respectively. In addition, a lot of aromatic amino acid residues existed around the six-membered ring, thus the molecular mobility of quinic acid was limited to some extent, resulting in enhancing the binding affinity to the three proteins. In summary, the data indicated that quinic acid could downregulate the level of core genes and had a potential binding activity with related target proteins in rhl system in QS signaling pathway, thus regulating effects on QS signaling pathways and subsequently resulting in a significant antibiofilm effect.

## Discussion

Pathogenic biofilms substantially increase resistance to conventional antibiotics. In the art, antibiofilm agents and QS inhibitors are proposed as alternatives of conventional antimicrobial chemotherapy to prevent the antibiotic resistance of bacteria [6, 34]. In the case of the study, an antibiotic-resistant PA named PA1803 was screened out with relatively high level of biofilm formation from the clinical PA strains. Subsequently, a series of QS-related phenotypic assays against PA1803 were further performed for 13 plant extracts. The results indicated that LJF could exert significant inhibitory effects on bacterial biofilm formation, mobility, toxin release and autoinducer release at sub-MICs, but not affecting bacterial growth. Moreover, in the biofilm-infection mouse model, LJF exerted an antibiofilm effect in vivo with lower colony counting and lower incidence of pathological changes such as abscess, bleeding and inflammation than those of control group.

To estimate active antibiofilm compounds in LJF, the antibiofilm activity of CA which was extensively reported as the main antibacterial component in LJF [35–38] was first tested. However, the results in comparative studies presented in Fig. 4a indicated that CA was not the most active ingredient responsible for antibiofilm effect of LJF in vitro. Moreover, the fact that CA was rapidly metabolized and was undetectable after entering the body suggested that CA was unlikely the active antibiofilm compound in the biofilm-infection mouse model. Further studies were conducted to discover more active ingredients exerting antibiofilm effect in LJF. Since many reports also indicated that the bioavailability of CA largely depended on its metabolism by the gut microflora [35, 39–43], it was speculated that some metabolites of CA were responsible for antibiofilm activity of LJF in vivo.

As one of the main active ingredients in many medicinal plants, CA appeared to provide high yields of microbial metabolites in vivo [44]. About 70% of CA reached the large intestine after oral administration [43, 45, 46] and thus the large intestine, especially the colon with rich flora, was the main metabolic absorption location for CA [47]. Most of CA, which was difficult in intestinal absorption and inclined to be affected by gut flora, was quickly hydrolyzed into caffeic acid and quinic acid by intestinal mucosa esterase and gut microflora, which made the in vivo bioavailability of CA per se very low and almost undetectable in plasma [40–42]. Subsequently, caffeic acid and quinic acid were further metabolized by the gut microflora (Fig. 4c left). Meanwhile, a small amount of CA, caffeic acid and quinic acid penetrated from gut barrier was further metabolized in the liver [48] (Fig. 4c right). In the study, it was speculated that the in vivo antibacterial and antibiofilm effects of CA were related to its microbial metabolites which were quickly metabolized after entering the body. Based on the comparative study on the antibacterial spectrum of CA and its metabolites, it was concluded that: (1) for most bacterial species, the metabolites such as dihydrocaffeic acid and gallic acid of CA could broaden antibacterial spectrums and enhance antibacterial activities; (2) for PA, the metabolites (eg, quinic acid) of CA likely enhanced the antibiofilm action other than the antibacterial action of CA, demonstrating better antibiofilm effect in vivo.

To verify the above two points, new experimental evidence was provided for the enhanced antibacterial activity of dihydrocaffeic acid and gallic acid and the effect of quinic acid in biofilm formation. In the comparative study on the antimicrobial activities of CA and its 13 metabolites in vitro, dihydrocaffeic acid and gallic acid showed the most potent antibacterial action (increased by 33 times against MSSA and MRSA). First, their effects on the integrity and the permeability of bacteria membrane were tested. It is well know that if the bacteria membrane is impaired or the permeability of bacterial cell membrane changes, it can be monitored by the release of cytoplasmic constituents of the cell. The amount of DNA and RNA released from the cytoplasm can be assessed through the absorbance at 260 nm. Similarly, the permeability of bacterial cell membrane can be monitored by the conductivity of bacteria liquid. Gallic acid has stronger antibacterial effect on the integrity of bacteria membrane. In the study, both dihydrocaffeic acid and gallic acid gradually caused the increase of the conductivity of bacterial liquids in interaction time, indicating that they can increase the permeability of the membrane.

Further, quinic acid at some sub-MICs significantly inhibited EPS secretion in biofilm formation and mature biofilm of PA1803. PA produced three key extracellular polysaccharides as main components of EPS: alginate, Pel and Psl, which determined the stability of the biofilm structure. Their synthesis and transport relied on the corresponding alginate, Psl and Pel biosynthetic systems [49–52]. Moreover, pslI and pslF related to psl synthesis were also decreased in PCR validation, reflecting the downrelation of these psl genes was involved in the inhibitory effect on EPS secretion in biofilm formation.

Meanwhile, the stability and toxicity of the metabolites CA of especially quinic acid were focused in the study. From the data of the stability of 13 metabolites of CA in vitro and quinic acid in vivo within 24 h (See Additional file 1: Tables S2, S3), no apparent degradation of quinic acid was found in both in vitro and in vivo conditions, reflecting quinic acid was relatively stable under physiological pH and enzymatic conditions. Also, in the test of the toxicity of quinic acid (0 to 1000 µg/mL) against GES-1(human gastric mucosal cell line) and Caco-2 (human intestinal cell line), no significant influence was observed (See Additional file 1: Fig. S1), reflecting that the doses of quinic acid were almost nontoxic to the epithelial cells of gastrointestinal tract. Thus oral administration was feasible. Similarly, the nontoxicity of quinic acid against HFF (human foreskin fibroblast) and THP-1 (human monocyte) indicated the possibility of dermal topical administration.

For PA, macrolides and fluoroquinolones have been extensively reported as effective antibiofilm agents [53–62]. Among the fluoroquinolones in these reports, levofloxacin is the most active, in particular against PA mature biofilms [55]. The synergistic antibiofilm effects of quinic acid at sub-MICs with two macrolides (clarithromycin and azithromycin) and levofloxacin were tested in the study. From the experimental results, it was found that the combined use of levofloxacin and quinic acid resulted in an enhanced therapeutic efficacy of levofloxacin in biofilm formation. However, quinic acid at sub-MICs and two macrolides (clarithromycin and azithromycin) did not show significant synergistic antibiofilm effect. In some reports, the combination of clarithromycin and levofloxacin was claimed effective in treating biofilm-associated chronic respiratory infection, which probably resulted from the activity of clarithromycin in removing the polysaccharide glycocalyx in or on bacterial biofilms [56]. Taking into consideration that quinic acid had inhibitory effect on the synthesis of extracellular polysaccharides in the case of the study, the mechanism of action was similar for the two macrolides and quinic acid. They destroyed the structure of extracellular polysaccharides in *Pseudomonas* biofilm matrix, making them or other antibacterial drugs (eg. levofloxacin) penetrate into the inner layer of bacterial biofilms. Thus, it was speculated that the combined use of macrolides and quinic acid likely showed limited synergistic antibiofilm effect and even competitively inhibitory effect.

Nevertheless, the mechanism of action of levofloxacin on biofilms was different from that of macrolides in the following 4 aspects: (1) electrostatic interference with the adhesion of bacteria and/or glycocalyx to the substratum, (2) activation or release of EPS-associated enzymes to disrupt the exopolysaccharide in the biofilm [63], (3) inhibition of the synthesis of bacterial nucleic acids, consequently reducing the amount of extracellular DNA, one of the most important compounds that increases the density and strength of EPS, and (4) bactericidal effect in the stationary phase of growth of bacteria [64]. It is clear from the above description that levofloxacin can affect multiple pathways and targets in the formation of bacterial biofilm which is possibly due to the synergistic antibiofilm effect of it with quinic acid.

Meanwhile, a comprehensive study for clarifying the underlying mechanism of antibiofilm activity of quinic acid, including the regulatory effects on QS-related genes and proteins, was also performed. GO analysis showed potential targets for antibiofilm activity of quinic acid against PA involved in multiple biological processes which were mostly relevant to locomotion, chemotaxis, movement and motility mediated by flagellum/cilium. Flagella, motility and chemotaxis are very important in the biofilm formation in some bacteria [65]. It was reported that flagellum deficient mutants showed poor adhesion ability on PVC, indicating that the physical attachment mediated by flagellum played important roles at the early stage of biofilm formation, including: (1) flagellum and cilia were important tools for bacteria in physical attachment. For instance, flagellum mediated chemotaxis can promote planktonic cells to migrate to the sites with sufficient nutrition on the surface, and sense the signals from attached cells; (2) flagellum mediated motility enabled bacteria to reach the surface at the very beginning and overcame the electrostatic repulsion between cells and the surface; (3) the flagellum mediated motility promoted the growth and extension of biofilm [66].

KEGG pathways analysis suggested that potential targets of antibiofilm activity of quinic acid against PA significantly enriched in the pathways of flagellar assembly, bacrerial chemotaxis, oxidative phosphorylation, ribosome, biofilm formation, cyanoamino acid metabolism and quorum sensing. Besides pathways such as flagellar assembly, bacrerial chemotaxis and quorum sensing involved in biofilm formation, cyanoamino acid metabolism and ribosome could induce a wide range of metabolism and synthesis of intracellular signal molecules and proteins including biofilm formation. Results of qRT-PCR suggested that LJF could significantly downregulate the level of rhlA, rhlR and rhlB in rhl system in QS signaling pathway. Computational modeling and interaction analysis results indicated that quinic acid fitted inside a pocket on the receptor structure by forming 5–10 hydrogen bonds with RhlA, RhlR and RhlB. The binding of quinic acid to RhlA, RhlR and RhlB receptor possibly interfered the binding of signal molecules to these receptors, resulting in transcriptional regulating effect on QS signaling pathways. In summary, target genes such as rhlA, rhlR and rhlB and related targets in rhl system in QS signaling were involved in antibiofilm action of quinic acid against drug-resistant PA.

## Conclusions

The study demonstrates that (1) the metabolites of CA such as quinic acid play an important role in antibiofilm action of LJF against drug-resistant PA in vitro and in vivo; (2) quinic acid can exert antibiofilm effect by regulating the transcriptional level of target genes, particularly by competitively inhibiting the binding of signaling molecules to target proteins in rhl system in QS signaling pathways related to biofilm formation. It is concluded that quinic acid can serve as a novel QS-based agent to prevent bacterial biofilm formation and treat related infections against clinical PA.

## Supplementary Information

**Additional file 1: Table S1.** The MICs of levofloxacin against 96 clinical isolates of PA. **Table S2.** The stability of 13 metabolites of CA in vitro within 24 hours. **Table S3**. The stability of quinic acid in vivo within 24 hours. **Fig.S1.** The effect of quinic acid on cell viability of normal cells.

## Data Availability

The datasets used in this study are available from the corresponding author upon reasonable request.

## Abbreviations

AMR: Antimicrobial resistance
LJF: *Lonicerae Japonicae Flos*
CA: Chlorogenic acid
QS: Quorum sensing
MIC: Minimum Inhibitory Concentration
sub-MICs: Sub-minimum inhibitory concentrations
AHLs: Acylated Homoserine Lactones
MSSA: Methicillin-sensitive *Staphylococcus aureus*
MRSA: Methicillin-resistant *Staphylococcus aureus*
qRT-PCR: Quantitative real-time polymerase chain reaction
DEG: Differentially Expressed Gene
GO: Gene Ontolog
KEGG: Kyoto Encyclopedia of Genes and Genomes
RPKM: Reads per Kilobase per Million Reads
