# Supplementary material for: Quinic acid: a potential antibiofilm agent against clinical resistant Pseudomonas aeruginosa
Source: Chin Med. 2021 Aug 6;16:72. doi: 10.1186/s13020-021-00481-8 (PMC8343939; doi:10.1186/s13020-021-00481-8)
Supplement: Supplementary file 1 — Additional file 1: Table S1. The MICs of levofloxacin against 96 clinical isolates of PA. Table S2. The stability of 13 metabolites of CA in vitro within 24 hours. Table S3. The stability of quinic acid in vivo within 24 hours. Fig.S1. The effect of quinic acid on cell viability of normal cells. [file 13020_2021_481_MOESM1_ESM.docx]

**Additional data:**

Table S1. The MICs of levofloxacin against 96 clinical isolates of PA

| Strain | MIC (mg/L) | Strain | MIC (mg/L) | Strain | MIC (mg/L) | Strain | MIC (mg/L) |
| --- | --- | --- | --- | --- | --- | --- | --- |
| Susceptible strains: n = 84 | | | | | | |  |
| PA1702 | 0.125 | PA1805 | 1 | PA1843 | 0.06 | PA1915 | 0.5 |
| PA1704 | 0.03 | PA1807 | 1 | PA1845 | 2 | PA1916 | 1 |
| PA1705 | 0.06 | PA1808 | 0.125 | PA1846 | 0.125 | PA1917 | 0.06 |
| PA1706 | 1 | PA1811 | 2 | PA1848 | 1 | PA1920 | 0.5 |
| PA1708 | 0.5 | PA1812 | 0.03 | PA1849 | 1 | PA1925 | 1 |
| PA1711 | 0.125 | PA1815 | 2 | PA1850 | 1 | PA1927 | 4 |
| PA1713 | 1 | PA1816 | 1 | PA1851 | 0.5 | PA1928 | 0.03 |
| PA1715 | 0.25 | PA1819 | 0.03 | PA1853 | 0.5 | PA1929 | 1 |
| PA1716 | 1 | PA1821 | 2 | PA1854 | 2 | PA1930 | 0.125 |
| PA1718 | 0.03 | PA1822 | 0.5 | PA1855 | 2 | PA1931 | 0.25 |
| PA1719 | 2 | PA1824 | 0.06 | PA1856 | 4 | PA1932 | 0.5 |
| PA1722 | 0.06 | PA1825 | 4 | PA1858 | 0.06 | PA1934 | 0.5 |
| PA1723 | 0.06 | PA1829 | 2 | PA1901 | 0.5 | PA1937 | 2 |
| PA1725 | 0.5 | PA1831 | 1 | PA1902 | 0.5 | PA1938 | 1 |
| PA1729 | 4 | PA1833 | 0.06 | PA1905 | 0.25 | PA1940 | 0.06 |
| PA1730 | 0.06 | PA1834 | 0.5 | PA1907 | 1 | PA1941 | 0.06 |
| PA1731 | 0.5 | PA1836 | 0.25 | PA1909 | 0.06 | PA1943 | 0.06 |
| PA1732 | 0.125 | PA1839 | 0.25 | PA1910 | 0.06 | PA1944 | 0.5 |
| PA1733 | 0.25 | PA1840 | 4 | PA1911 | 2 | PA1946 | 1 |
| PA1801 | 0.5 | PA1841 | 0.5 | PA1912 | 1 | PA1947 | 0.25 |
| PA1804 | 0.25 | PA1842 | 2 | PA1913 | 0.5 | PA1949 | 1 |
| drug-resistant strains: n=12 | | | | | | | |
| PA1701 | 16 | PA1727 | 8 | PA1817 | 8 | PA1904 | 16 |
| PA1709 | 8 | PA1802 | 8 | PA1852 | 16 | PA1919 | 16 |
| PA1721 | 8 | PA1803 | 8 | PA1857 | 16 | PA1935 | 8 |

The MICs were determined as 90% inhibition of planktonic growth with the highest frequencies for each strain. Values are averages from three independent experiments performed in sextuplicate, with ranges in parentheses.

Table S2 The stability of 13 metabolites of CA in vitro within 24 hours

| Compound | Peak areas | | | | | | RSD (%) |
| --- | --- | --- | --- | --- | --- | --- | --- |
|  | 0H | 2H | 4H | 6H | 12H | 24H |  |
| Gallic acid | 3030.1 | 3043.3 | 2986.6 | 3062.5 | 3090.3 | 3077.3 | 1.22 |
| P-hydroxybenzoic acid | 3426.2 | 3410.3 | 3450.6 | 3459.7 | 3442 | 3430.9 | 0.52 |
| Benzoic acid | 1946.3 | 1945.3 | 1945.3 | 1944.5 | 1943.8 | 1944.6 | 0.04 |
| P-coumaric acid | 12157.4 | 11947.3 | 12059.4 | 12175.7 | 12413.4 | 12076.1 | 1.30 |
| Chlorogenic acid | 4052.5 | 4060 | 4069.2 | 4064.2 | 4060 | 4065.7 | 0.14 |
| Dihydrocaffeic acid | 2671.1 | 2675 | 2671.8 | 2667.5 | 2673.8 | 2670.5 | 0.10 |
| Hippuric acid | 1197.2 | 1199.6 | 1201.9 | 1207.5 | 1206.3 | 1209.8 | 0.41 |
| Cinnamic acid | 9196.8 | 9195.3 | 9208.5 | 9201.3 | 9226.7 | 9259.3 | 0.27 |
| Vanillic acid | 12702 | 12722.3 | 12713.7 | 12700.8 | 12682.7 | 12742.6 | 0.16 |
| Ferulic acid | 8078.1 | 8132.5 | 8132.8 | 8146.1 | 8085.1 | 8069.5 | 0.41 |
| Butyric acid | 12525.4 | 12548.8 | 12452.3 | 12485.9 | 12547 | 12467.8 | 0.33 |
| Quinic acid | 1021.8 | 1018.6 | 1020.1 | 1029.5 | 1038.2 | 1036.7 | 0.84 |
| Shikimic acid | 2021.1 | 2042.3 | 2086.7 | 2062.5 | 2090.1 | 2077.8 | 1.32 |

Table S3 The stability of quinic acid in vivo within 24 hours

| Compound | Peak areas | | | | | | RSD (%) |
| --- | --- | --- | --- | --- | --- | --- | --- |
|  | 0H | 2H | 4H | 6H | 12H | 24H |  |
| Quinic acid | 934512 | 1007391 | 1004372 | 1160787 | 1172793 | 1184353 | 10.00 |


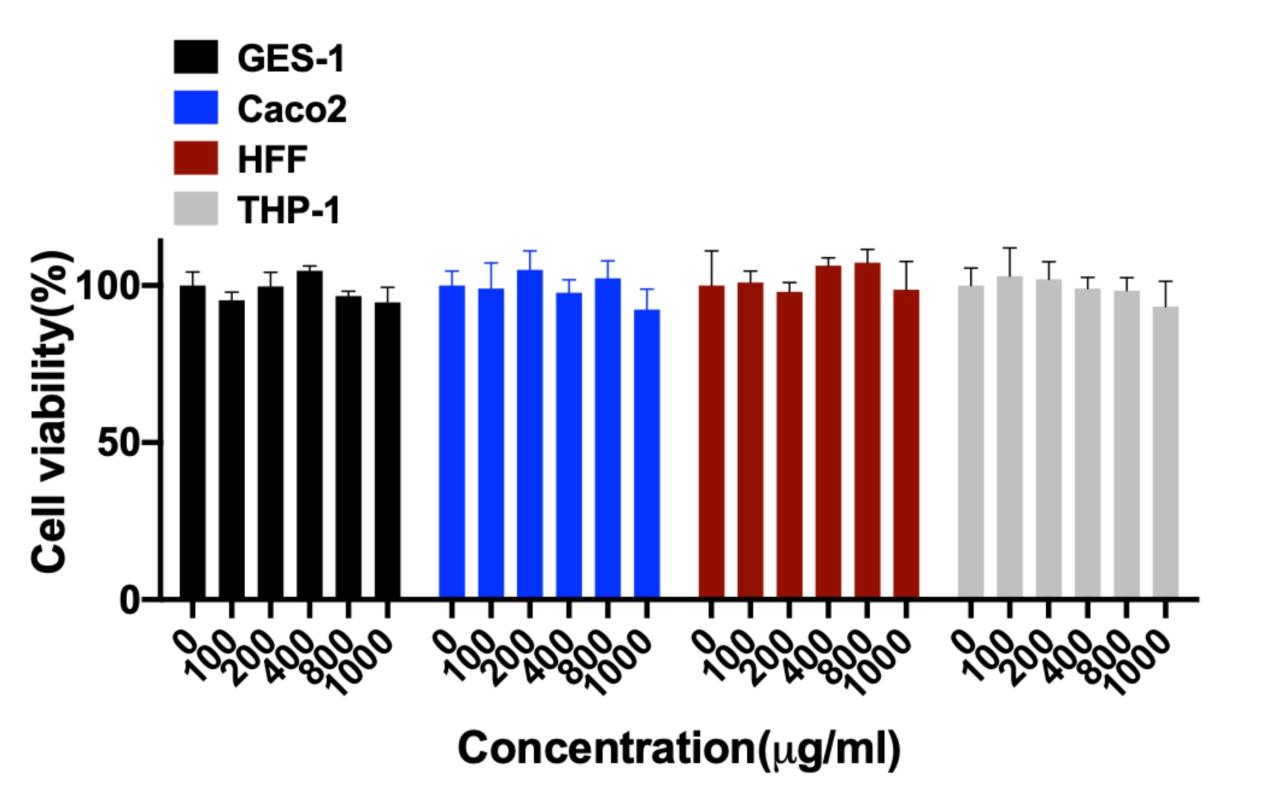


Fig.S1 The effect of quinic acid on cell viability of normal cells.
